# Isolation of multidrug-resistant *Escherichia coli* and *Salmonella* spp. from sulfonamide-treated diarrheic calves

**DOI:** 10.14202/vetworld.2022.2870-2876

**Published:** 2022-12-16

**Authors:** Mohammad Amdadul Haque, Muhammad Tofazzal Hossain, Md. Shafiqul Islam, Md. Zahorul Islam, Purba Islam, Sourendra Nath Shaha, Mahmudul Hasan Sikder, Kazi Rafiq

**Affiliations:** 1 Department of Pharmacology, Bangladesh Agricultural University, Mymensingh, Bangladesh; 2 Department of Microbiology and Hygiene, Bangladesh Agricultural University, Mymensingh, Bangladesh; 3 Directorate of Livestock Services, Farmgate, Dhaka, Bangladesh

**Keywords:** antimicrobial resistance, calf, diarrhea, *Escherichia coli*, multidrug-resistant, *Salmonella* spp

## Abstract

**Background and Aim:**

The bovine industry is threatened by one of the most serious and deadly enteric diseases, calf diarrhea, particularly in developing nations like Bangladesh. In this context, bacterial resistance to antimicrobial drugs and its detrimental consequences have become a critical public health issue that is difficult to address globally. This study aimed to isolate and identify *Escherichia coli* and *Salmonella* spp. with their antibiogram and antibiotic resistance gene detection from sulfonamide-treated diarrheic calves.

**Materials and Methods:**

Twelve diarrheic calves suffering from calf diarrhea in a dairy farm were selected and a total of 36 fecal samples were aseptically collected directly from rectum before, during, and at the end of treatment for each calf to determine the total viable count, total *E. coli* count and total *Salmonella* count. A polymerase chain reaction was used for the specific detection of *E. coli* and *Salmonella* genus targeting *fliC* and *invA* genes, respectively. Antibiotic sensitivity test of the isolated *E. coli* and *Salmonella* spp. were performed by the disk diffusion method for eight commonly used antibiotics.

**Results:**

A total of 36 *E. coli* (100%) and 12 *Salmonella* spp. (33%) were isolated from the samples and were confirmed by polymerase chain reaction. Total viable count was found to be ranged from 35 × 10^7^ to 99 × 10^10^ colony-forming unit (CFU)/g fecal sample before starting sulfonamide treatment, 34 × 10^5^ to 25 × 10^10^ CFU/g during treatment with sulfonamide, and 48 × 10^3^ to 69 × 10^10^ CFU/g immediately after completion of sulfonamide treatment. Total *E. coli* count was found to be ranged from 4 × 10^4^ to 36 × 10^10^ CFU/g, 24 × 10^4^ to 23 × 10^8^ CFU/g, and 13 × 10^4^ to 85 × 10^10^ CFU/g, whereas total *Salmonella* count was found to be ranged from 16 × 10^6^ to 18.5 × 10^11^ CFU/g, 15 × 10^4^ to 44 × 10^7^ CFU/g, and 13.2 × 10^5^ to 21 × 10^10^ CFU/g fecal sample before starting sulfonamide treatment, during treatment with sulfonamide immediately after completion of sulfonamide treatment, respectively. The *in vitro* antibiotic sensitivity test showed that all the *E. coli* and *Salmonella* spp. isolated from diarrheic calves (100%) contained multidrug-resistant (MDR) phenotypes. *Escherichia coli* isolates were found 100% resistant to amoxicillin (AMX), cefuroxime, cephalexin (CN), erythromycin (ERY), and tetracycline (TET); whereas 94.4%, 86.1%, and 77.8% isolates were resistant to doxycycline (DOX), moxifloxacin (MOF), and gentamycin (GEN), respectively. In case of *Salmonella* isolates, all were found 100% resistant to AMX, CN, and ERY; whereas 91.7% of resistance was observed for DOX, MOF, cefuroxime, GEN, and TET. Based on the molecular screening of the antibiotic resistance genes, *tet*A gene was present in 83.3% of the isolated *E. coli* and 75% of the isolated *Salmonella* strains, whereas 83.3% *E. coli* and 79.2% *Salmonella* isolates contained *bla*TEM gene.

**Conclusion:**

These findings suggest that MDR *E. coli* and *Salmonella* spp. might be responsible for calf scouring, which is challenging to treat with antibiotics or sulfonamide drugs alone. Therefore, it is important to check the antibiotic sensitivity pattern to select a suitable antibiotic for the treatment of calf scoring. A suitable antibiotic or combination of an antibiotic and sulfonamide could be effective against *E. coli* and *Salmonella* spp. responsible for calf scouring.

## Introduction

Calf diarrhea is one of the most important devastating enteric problems that threaten the bovine industry worldwide [1], with high morbidity and mortality rates, especially in a developing country like Bangladesh [2]. In Bangladesh, calf diarrhea remains the most frequently recorded clinical concern in livestock sector [3]. Bacteria (*Salmonella* spp., *Escherichia* *coli*, and *Clostridium perfringes*), protozoa (*Cryptosporidium parvum*), and viruses (coronavirus and rotavirus) may cause diarrhea in calves [4–7] alone or in combination with other associated pathogens [8]. Among these agents, *E. coli* and *Salmonella* spp. are the most economically important pathogens [9] and are frequently associated with calf diarrhea in Bangladesh [2]. To treat bacterial diarrhea in calves, a course of antimicrobial therapy is required. However, antimicrobials are used indiscriminately and in low doses for preventive and curative purposes worldwide in calf feed to prevent the major economic loss caused by the bacteria [10]. Sulfonamide has been used widely to treat bacterial and protozoal infections over several decades. In addition, sulfonamides are commonly used alone or in combination with trimethoprim or with other antibiotics for both prophylactic and treatment of calf diarrhea [11]. Although sulfonamides are highly effective against calf diarrhea caused by both *E. coli* and *Salmonella* spp. [12]; however, persistent and indiscriminate use of antimicrobials, incomplete course, and lack of maintenance of withdrawal period may lead to the development of a new generation of virulent and resistant bacterial strains that may reduce its efficacy or effectiveness. In this regard, field veterinarian from different parts of Bangladesh is claiming the ineffectiveness of sulfonamide therapy in calf diarrhea. Antimicrobial drug resistance to bacteria and its adverse consequence has become a serious public health concern worldwide [13]. Antimicrobial resistance (AMR) has been frequently observed in *Salmonella* spp. and *E. coli* species, especially in pre-weaned dairy calves [4]. In these regards, several studies have been done for the isolation, identification, antimicrobial sensitivity testing, and characterization of the resistant genes from both *E. coli* and *Salmonella* spp. in home and abroad [14–16]. However, to the best of our knowledge, there are no data available regarding the isolation and identification of *E. coli* and *Salmonella* spp. with their antimicrobial sensitivity pattern from sulfonamide-treated diarrheic calves time-dependently in Bangladesh.

Therefore, this study was carried out to isolate *E. coli* and *Salmonella* spp. with their antibiotic sensitivity pattern and antibiotic resistance genes during the course of sulfonamide treatment in diarrheic calves. Our present study findings highlighted the detection of multidrug-resistant (MDR) *E. coli* and *Salmonella* spp. from sulfonamide-treated diarrheic calves, which is difficult to treat clinically with sulfonamide or antibiotic singly.

## Materials and Methods

### Ethical approval

All experimental procedures were performed according to the guidelines for the care and use of animals as described by Animal Welfare and Experimentation Ethics Committee, Bangladesh Agricultural University, Mymensingh-2202 (Approval number: AWEEC/BAU/2018[11]).

### Study period and location

The study was conducted from October 2018 to March 2019 in collaboration with the Department of Pharmacology, and Department of Microbiology and Hygiene, Bangladesh Agricultural University, Mymensingh.

### Collection of samples

Twelve diarrheic calves (1–3 months of age) suffering from calf scours (calf diarrhea) in a dairy farm located at Trishal Upazila, Mymensingh district, Bangladesh were selected and a total of 36 fecal samples were aseptically collected at three different time points directly from rectum basis on their previous history of treatment failure against calf diarrhea treated with sulfonamide, conventional antibiotics, or their combinations. The calves were divided into three groups (C, T and TC), where “C” represents samples (C1–C12) collected from diarrheic calves before treatment with sulfonamide, “T” represents samples (T1–T12) collected during the treatment with sulfonamide and “TC” for samples collected immediately after completion of sulfonamide treatment. The samples were transferred to sterile polythene zip-lock bags after collection and brought to the bacteriological laboratory, Department of Microbiology and Hygiene, Bangladesh Agricultural University, Mymensingh, in a transport box containing ice.

### Enumeration of bacterial load

One gram of each feces sample was used to determine the total viable count (TVC), total *E. coli* count (TEC), and total *Salmonella* count (TSC) according to the previously published methods [17, 18]. Briefly, a total of 900 μL of phosphate buffer solution was taken in eight Eppendorf tubes, and 100 μL suspension was used to prepare ten serial-fold dilution of each content. Then, 10 μL from each dilution was dropped on plate count agar (HiMedia, India) for TVC, on Eosin-Methylene-Blue (EMB) agar (HiMedia) for TEC, and on Salmonella-Shigella (SS) agar (HiMedia) for TSC and were overnight incubated in a bacteriological incubator at 37°C. Colonies for suitable dilution were counted, and TVC, TEC, and TSC were calculated.

### Isolation and identification of *E. coli* and *Salmonella* spp.

All collected feces samples were enriched in nutrient broth followed by overnight incubation at 37°C. The enriched culture of each sample was then streaked onto EMB and SS agar media for the isolation of *E. coli* and *Salmonella* spp., respectively. A suspected single colony was further streaked onto same media to obtain pure cultures [19]. In addition, Gram’s staining was also performed for morphological identification of *E. coli* and *Salmonella* spp. isolated from fecal samples [19, 20].

### Molecular detection

Primers and polymerase chain reaction (PCR) conditions used for the specific detection of *E. coli* and *Salmonella* genus targeting 16S rRNA and *invA* genes, respectively, are presented in Table-1 [20–22]. For PCR, genomic DNA was extracted from *E. coli* and *Salmonella* spp. by simple boiling method as described previously by Hossain *et al*. [23]. Briefly, a pure colony of each isolate was inoculated into the broth. After overnight incubation, 1 mL of cultural broth was centrifuged at 10,000 rpm for 3 min. The supernatant was discarded and suspended with 100 μL distilled water, boiling for 20 min followed by cold shock for about 7 min and then centrifuged at 10,000 rpm for 10 min. Finally, supernatant was collected, stored and used as DNA template for PCR. The PCR was performed in an applied Biosystem Thermocycler (Thermo Fisher Scientific, USA) in a total volume of 25 μL reaction mixture with 12.5 μL master mixture 2× (Promega, USA), 3 μL (50 ng) genomic DNA, 1 μL of each primer, and 7.5 μL nuclease-free water. Amplified products were analyzed by electrophoresis in 1.5% of agarose gel, stained in ethidium bromide, and finally visualized under an ultraviolet transilluminator (Biometra, Germany). The size of PCR amplicons was assessed using a 100 bp DNA ladder (Promega).

**Table-1:** Primer sequences and PCR conditions.

Target gene	Primer sequences	Amplified segment (bp)	Primary denaturation	Amplification (30–34 cycles)			Final extension	Reference

				Secondary denaturation	Annealing	Extension		
*16SrRNA* for *E. coli*	F: CCCCCTGGACGAA GACTGAC R: ACCGCTGGCAACA AAGGATA	401	95°C 5 min	94°C 30 s	57°C 90 s	72°C 90 s	72°C 10 min	[21]
*invA* for *Salmonella*	F: ATCAGTACCAGTC GTCTTATCTTGAT R: TCTGTTTACCGGG CATACCAT	211	94°C 5 min	94°C 30 s	52°C 1 min	72°C 45 s	72°C 5 min	[20]
*bla*TEM	F: CATTTCCGTGTCG CCCTTAT R: TCCATAGTTGCCT GACTCCC	793	95°C 5 min	95°C 1 min	52°C 1 min	72°C 1 min	72°C 7 min	[22]
*tet*A	F: GGTTCACTCGAAC GACGTCA R: CTGTCCGACAAGT TGCATGA	577	95°C 5 min	95°C 1 min	52°C 1 min	72°C 1 min	72°C 10 min	[20]

PCR=Polymerase chain reaction, *E. coli*=*Escherichia coli*

### Antibiotic sensitivity test

Antibiotic sensitivity test of the isolated *E. coli* and *Salmonella* spp. was performed by disk diffusion method [24]. Freshly grown isolates having a concentration equivalent to 0.5 McFarland standards were spread on Mueller-Hinton agar media (HiMedia) using a sterile cotton swab and eight commonly used antibiotics of HiMedia, namely, amoxicillin (AMX, 30 μg/disc), gentamycin (GEN, 10 μg/disc), tetracycline (TET, 30 μg/disc), erythromycin (ERY, 15 μg/disc), doxycycline (DOX, 30 μg/disc), moxifloxacin (MOF, 5 μg/disc), cephalexin (CN, 30 μg/disc), and cefixime (5 μg/disc) were placed on the media. All results of antibiotic susceptibility for *E. coli* and *Salmonella* spp. were interpreted according to the guidelines provided by Clinical and Laboratory Standards Institute [25].

### Molecular detection of TET and beta-lactams resistant genes

The presence of TET-resistant *tetA* and beta-lactam-resistant *bla*TEM genes in the isolated *E. coli* and *Salmonella* spp. was screened by PCR using the mixture conditions as described by Tawyabur *et al*. [20] and Walker *et al*. [22], respectively. Primers with PCR conditions used for the specific detection of *tet*A and *bla*TEM genes are presented in Table-1.

### Statistical analysis

All the collected data were analyzed with the help of GraphPad Prism 6 (2365 Northside Dr. Suite 560. San Diego, CA 92108). The mean differences between before, during, and after the treatments were determined by a one-way analysis of variance followed by Bonfferoni *post hoc* test [26].

## Results and Discussion

### Enumeration of TVC, TEC, and TSC from diarrheic calves

The total viable count was found to be ranged from 35 × 10^7^ colony-forming unit (CFU)/g to 99 × 10^10^ CFU/g fecal sample before starting sulfonamide treatment, 34 × 10^5^ CFU/g to 25 × 10^10^ CFU/g during the treatment with sulfonamide, and 48 × 10^3^ CFU/g to 69 × 10^10^ CFU/g immediately after completion of sulfonamide treatment. The lowest and highest TVC was found in calf of sample C-9 and C-2, sample T-12 and T-3, and sample TC-11 to TC-9, respectively (Table-2). Total *E. coli* count was found 4 × 10^4^ CFU/g to 36 × 10^10^ CFU/g, 24 × 10^4^ CFU/g to 23 × 10^8^ CFU/g, and 13 × 10^4^ CFU/g to 85 × 10^10^ CFU/g in samples before starting sulfonamide treatment, during treatment with sulfonamide, and immediately after completion of sulfonamide treatment, respectively (Table-2). total *Salmonella* count was also found 16 × 10^6^ CFU/g to 18.5 × 10^11^ CFU/g, 15 × 10^4^ CFU/g to 44 × 10^7^CFU/g, and 13.2 × 10^5^ CFU/g to 21 × 10^10^ CFU/g in samples before starting sulfonamide treatment, during the treatment with sulfonamide, and immediately after completion of sulfonamide treatment, respectively (Table-2). Variation in results of TVC, TEC, and TSC indicates that sulfonamide is not always effective for diarrheic calves. In this regard, Klaus *et al*. [27] reported that sulfonamides used for the treatment of neonatal calves with diarrhea were effective in their clinical improvement, but systemic therapy with sulfonamide plus antibiotics provided better performance, with better weight gain and body development.

**Table-2:** Results of bacterial load in feces collected from diarrheic calf at three different time points (before starting, during, and immediately after completion of sulfonamide treatment).

Calf ID	Bacterial load (CFU/g)								

	TVC			TEC			TSC		
		
	C: Before	T: During	TC: After	C: Before	T: During	TC: After	C: Before	T: During	TC: After
1	60 × 10^10^	14 × 10^8^	26 × 10^6^	42 × 10^6^	41 × 10^7^	16 × 10^5^	16 × 10^6^	44 × 10^7^	13.2 × 10^5^
2	99 × 10^10^	21 × 10^6^	11 × 10^6^	22 × 10^5^	22 × 10^5^	22 × 10^5^	-	-	-
3	81 × 10^9^	25 × 10^10^	33 × 10^8^	22 × 10^7^	22 × 10^7^	17 × 10^8^	21 × 10^7^	18 × 10^7^	82 × 10^6^
4	41 × 10^9^	29 × 10^9^	36 × 10^10^	35 × 10^6^	35 × 10^6^	22 × 10^7^	-	-	-
5	85 × 10^8^	25 × 10^6^	18 × 10^8^	10 × 10^5^	10 × 10^5^	16 × 10^7^	-	-	-
6	20 × 10^9^	27 × 10^9^	53 × 10^9^	4 × 10^4^	24 × 10^5^	13 × 10^4^	-	-	-
7	38 × 10^10^	72 × 10^9^	17 × 10^10^	22 × 10^10^	24 × 10^4^	21 × 10^10^	18.5 × 10^11^	15 × 10^4^	21 × 10^10^
8	13.9 × 10^10^	22 × 10^9^	72 × 10^9^	61 × 10^9^	70 × 10^4^	37 × 10^9^	45 × 10^10^	20 × 10^4^	78 × 10^8^
9	35 × 10^7^	12 × 10^8^	69 × 10^10^	8 × 10^8^	5 × 10^6^	57 × 10^10^	-	-	-
10	39 × 10^10^	25 × 10^8^	29 × 10^6^	36 × 10^10^	10 × 10^8^	11 × 10^5^	-	-	-
11	20 × 10^8^	33 × 10^9^	48 × 10^3^	14 × 10^8^	23 × 10^8^	33 × 10^10^	-	-	-
12	22 × 10^10^	34 × 10^5^	11.3 × 10^11^	18 × 10^7^	7 × 10^5^	85 × 10^10^	-	-	-

TVC=Total viable cell count, TEC=Total *E. coli* count, TSC=Total salmonella count, CFU=Colony-forming unit

### Isolation and identification of *E. coli* and *Salmonella* spp. from diarrheic calves

**Figure-1:**
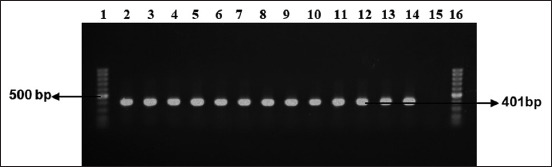
Polymerase chain reaction assay for the amplification of species-specific *16S rRNA* (401 bp) gene from *Escherichia coli*. Lanes 1 and 16: 100 bp ladder, L14: positive control, L15: negative control, and Lanes 2–13: *E. coli* isolates from a diarrheic calf.

**Figure-2:**
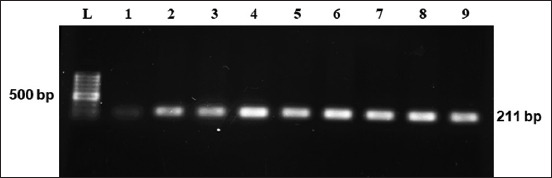
Polymerase chain reaction assay for the amplification of genus-specific *invA* (2101 bp) gene from *Salmonella* spp. Lane L: 100 bp ladder, Lanes 1–9: *Salmonella* isolates from a diarrheic calf.

Several enteric pathogens are responsible for causing neonatal diarrhea [27]. In this study, 36 *E. coli* (100%) and 12 *Salmonella* spp. (33%) were isolated and detected in 36 collected feces samples regardless of the collecting time. Moreover, 12 *Salmonella* spp. were isolated from 36 fecal samples collected at three different times (before, during, and after) of treatment with sulfonamide and identified by cultural and staining properties followed by PCR for confirmation (Figures-1 and 2). Isolation rate of *E. coli* from diarrheic calves in this study has a similarity with the findings of Gupta *et al*. [28] and Diwakar *et al*. [6]. Dark blue-black colonies of *E. coli* with metallic green sheen were found on EMB agar media, and raised, pinhead, round, or circular, black-centered colonies of *Salmonella* spp. were found on SS agar media. Gram-negative, pink-colored, single, or paired short plump rod-shaped appearance was observed in Gram’s staining both for suspected *E. coli* and *Salmonella*. All the culture-positive *E. coli* and *Salmonella* spp. were confirmed by PCR and positive bands appeared at 401 bp and 211 bp for *E. coli* and *Salmonella* spp., respectively (Figures-1 and 2).

### Occurrence of MDR *E. coli* and *Salmonella* spp. in diarrheic calves

The results of the antibiotic sensitivity test showed that all the *E. coli* and *Salmonella* spp. isolated from diarrheic calves (100%) showed MDR pattern (Table-3). It was found that 100% *E. coli* isolates were resistant to AMX, cefuroxime, CN, ERY, and TET; whereas 94.4%, 86.1%, and 77.8% isolates were resistant to DOX, MOF, and GEN, respectively (Table-3). In case of *Salmonella* isolates, all were found 100% resistant to AMX, CN, and ERY, whereas 91.7% of resistance was observed for DOX, MOF, cefuroxime, GEN, and TET (Table-3). Based on previously published evidence for the oral administration of these antimicrobial agents, Constable [29] recommended only AMX for the treatment of calf diarrhea. However, in this present study, all the isolates were found resistant to AMX that means it was not effective for these sick calves. Ansari *et al*. [14] also reported similar type of findings where 100% resistance was also observed against AMX. Gupta *et al*. [28] found that 83.33% *E. coli* isolates in their study were MDR, whereas all the *E. coli* and *Salmonella* spp. isolates of this study were MDR. Gentamicin was found to be relatively sensitive to the isolates compared to other antibiotics used in this study. Diwakar *et al*. [6] reported that GEN was the most effective antibiotic in case of calf diarrhea and highly sensitive for *E. coli*, *Shigella*, *Edwardsiella*, *Salmonella*, and *Klebsiella* as well as *Proteus* isolates recovered from cases of calf diarrhea. The presence of MDR *E. coli* and *Salmonella* spp. is documented as important public health hazards worldwide. Consequently, hospital costs for the treatment of both humans and livestock become expensive and would definitely prolong treatment duration time.

**Table-3:** Results of antimicrobial susceptibility testing for multidrug resistance of *E. coli* and *Salmonella* spp. isolated from diarrheic calves.

Number of isolates (n = 48)			

Antibiotics used	*E. coli* (n = 36)	*Salmonella* spp. (n = 12)	Overall resistance (%)
Amoxicillin	36	12	48 (100)
Cefuroxime	36	11	47 (97.9)
Cephalexin	36	12	48 (100)
Doxycycline	34	11	45 (93.8)
Erythromycin	36	12	48 (100)
Gentamycin	28	11	29 (60.4)
Moxifloxacin	31	11	42 (87.5)
Tetracycline	36	11	47 (97.9)

*E. coli*=*Escherichia coli*

### Detection of *tet*A and *bla*TEM genes in the isolates

**Figure-3:**
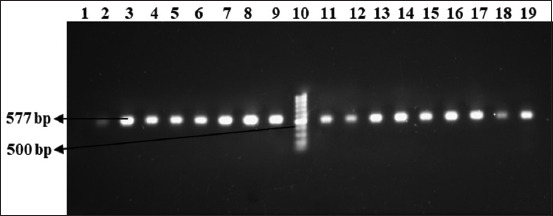
Amplification of *tet*A (577 bp) gene in isolated *Escherichia coli* and *Salmonella* spp. Lane 10: 100-bp DNA ladder, Lanes 1–9: Amplified product of DNA sample of *Escherichia coli*, and Lane 11–19: Amplified product of DNA sample of *Salmonella* spp.

**Figure-4:**
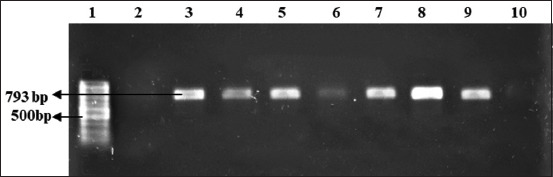
Amplification of *bla*TEM (793 bp) gene in isolated *Escherichia coli* and *Salmonella* spp. Lane 1: 100-bp DNA ladder, Lanes 2–5: Amplified product of DNA sample of *Escherichia coli*, and Lanes 6–10: Amplified product of DNA sample of *Salmonella* spp.

Based on the molecular screening of the antibiotic resistance genes, *tet*A gene was present in 83.3% of the isolated *E. coli* and 75% of the isolated *Salmonella* strains (Figure-3), whereas 83.3% *E. coli* and 79.2% *Salmonella* isolates contained *bla*TEM gene (Figure-4). The finding on *tet*A gene in this present study is compatible with the results of Hafez [30] and Liao *et al*. [31]; the active efflux is still the primary mechanism underlying *E. coli* resistance to TET. The findings on *bla*TEM also have similarities with the results of Hafez [30] and Rahman *et al*. [32].

Therefore, it is important to check the antibiotic sensitivity pattern to select a suitable antibiotic for the treatment of calf scoring. A suitable antibiotic or combination of an antibiotic and sulfonamide could be effective against *E. coli* and *Salmonella* spp. responsible for calf scouring. The anticipated data suggested the judicial use of antimicrobials, measurement to preserve antimicrobials’ effectiveness and suitable antimicrobials treatment strategies are necessary to control calf scouring which will definitely help to prevent antibiotic resistance.

## Limitations of the study

This study has several limitations, such as the sampling area that was limited to a dairy farm. Further details study with larger samples size from various dairy farms in Bangladesh is needed. Details of further phenotypic and genotypic analysis in a wider range with 16S rRNA sequence profiling of these isolates would definitely help the scientists in this field to combat AMR as well as to stop the spreading of MDR foodborne pathogens to humans.

## Conclusion

This study findings indicate a high frequency of AMR among *E. coli* and *Salmonella* spp. isolated from sulfonamide-treated diarrheic calves. *Escherichia coli* and *Salmonella* spp. are the important causes of calf diarrhea which cannot be managed by the use of sulfonamide drugs or antibiotics alone. For the quick recovery of the diarrheal calves, sulfonamide drugs in combination with antibiotics such as GEN may be beneficial. The results of this study will undoubtedly assist veterinarians in choosing the best treatment strategies against calf diarrhea that will help reduce MDR bacteria and fight against AMR.

## Authors’ Contributions

MAH and MTH: Performed all the experiments and prepared the draft of the manuscript; KR and MTH: Conceptualization, designed and supervised the research, revised, and finalized the draft of the manuscript; KR, MSI, MZI, PI, and MHS: Did the statistical analysis and prepared the graphs. MAH, SNS, and MTH: Revised the manuscript and prepared the graphs and tables. All authors have read and approved the final manuscript.
